# Supplementary material for: “Ghost” Fragment Ions in Structure and Site-Specific Glycoproteomics Analysis
Source: Anal Chem. 2023 Jun 29;95(27):10145–8. doi: 10.1021/acs.analchem.3c02207 (PMC10339278; doi:10.1021/acs.analchem.3c02207)
Supplement: Supplementary file 1 — ac3c02207_si_001.pdf [file ac3c02207_si_001.pdf]

Supporting Information

“Ghost” fragment ions in structure and site-specific glycoproteomics analysis

Diana Campos[a*], Michael Girgis[b], Qiang Yang[c], Guanghui Zong[d], Radoslav Goldman,[e,f], Lai-Xi Wang[d], Miloslav Sanda,[a,e,f*]

[a]Max-Planck-Institut fuer Herz- und Lungenforschung, Ludwigstrasse 43, 61231 Bad Nauheim, Germany; [b]Department of Bioengineering, College of Engineering and Computing, George Mason University, Fairfax, VA 22030, USA; [c]GlycoT Therapeutics, College Park, MD 20742, USA, [d]Department of Chemistry and Biochemistry, University of Maryland, College Park, MD 20742, USA; [e]Department of Oncology, Lombardi Comprehensive Cancer Center, Georgetown University, Washington, D.C 20057, USA; [f]Clinical and Translational Glycoscience Research Center, Georgetown University, Washington, D.C., 20057, USA.

*Corresponding author email: Miloslav.Sanda@mpi-bn.mpg.de; tel: +49 (0)6032 705-1760, Diana.Campos@mpi-bn.mpg.de, +49 (0)6032 705-1760

Contents:

1. Materials and Methods
2. Figure S1-S6
3. References

MATERIALS AND METHODS

Synthesis of 13C-Labeled IgG1-Fc Glycopeptides

IgG1-Fc glycopeptides were synthesized and purified as previously published by us [1, 2]. After lyophilization, the synthesized glycopeptides were weighed on an accurate balance and further quantitated by analytic HPLC. In the following procedures, we used the IgG1 glycopeptide labelled standars with G1F, G2F, SG2F and S2G2F structures with extended sequence AKTKPREEQYNSTYRVVS.

Glycopeptide Analysis by a Nano-LC-MS/MS-PRM workflow

2 µg of each IgG1 glycopeptide stable Isotope labeled standards was dissolved in 20µl of 50mM AmBic buffer. The glycopeptide mixture was reduced by 5mM DTT at 56°C and alkylated by 15mM IAA for 30min. Peptide mixture was digested by trypsin in a ratio 1:50 at 37°C overnight. Glycopeptides were separated by capillary reverse phase (C18) nano-chromatography on in-house packed silica emitter tip (15cm x 75 µm) using Easy Nano chromatographic system (Thermo). Glycopeptides were separated at 0.3 µL/min as follows: starting conditions 2% ACN, 0.1% formic acid; 0−35 min, 2− 50% ACN, 0.1% formic acid; 35−42 min, 50−90% ACN, 0.1% formic acid; 42−47 min 90% ACN, 0.1% formic acid followed by equilibration to starting conditions for additional 3 min. Q-exactive HF mass spectrometer (Thermo) was used for MS and MS/MS analysis. Fragmentation spectra were recorded at 30 000 resolving power. 100 pg of tryptic glycopeptide mixture containing G1F, G2F, SG2F and S2G2F structures was injected on column. Separated Parallel Reaction Monitoring (PRM) methods with transition lists with Normalized collision energy (NCE) range 10-60 using 5stpes were created for each glycopeptide standard. Two charge states (+2 and +3) were subjected to fragmentation analysis. Qual-browser software was used for qualitative and quantitative data processing. 3 replicates of each standard were measured.

Xcalibur (Thermo) software was used for quantitative data processing. Processing methods were created for ion extraction from each PRM transition. Area of integrated peak was used for further data processing. Further data processing and graphing was carried out in Microsoft Excel.

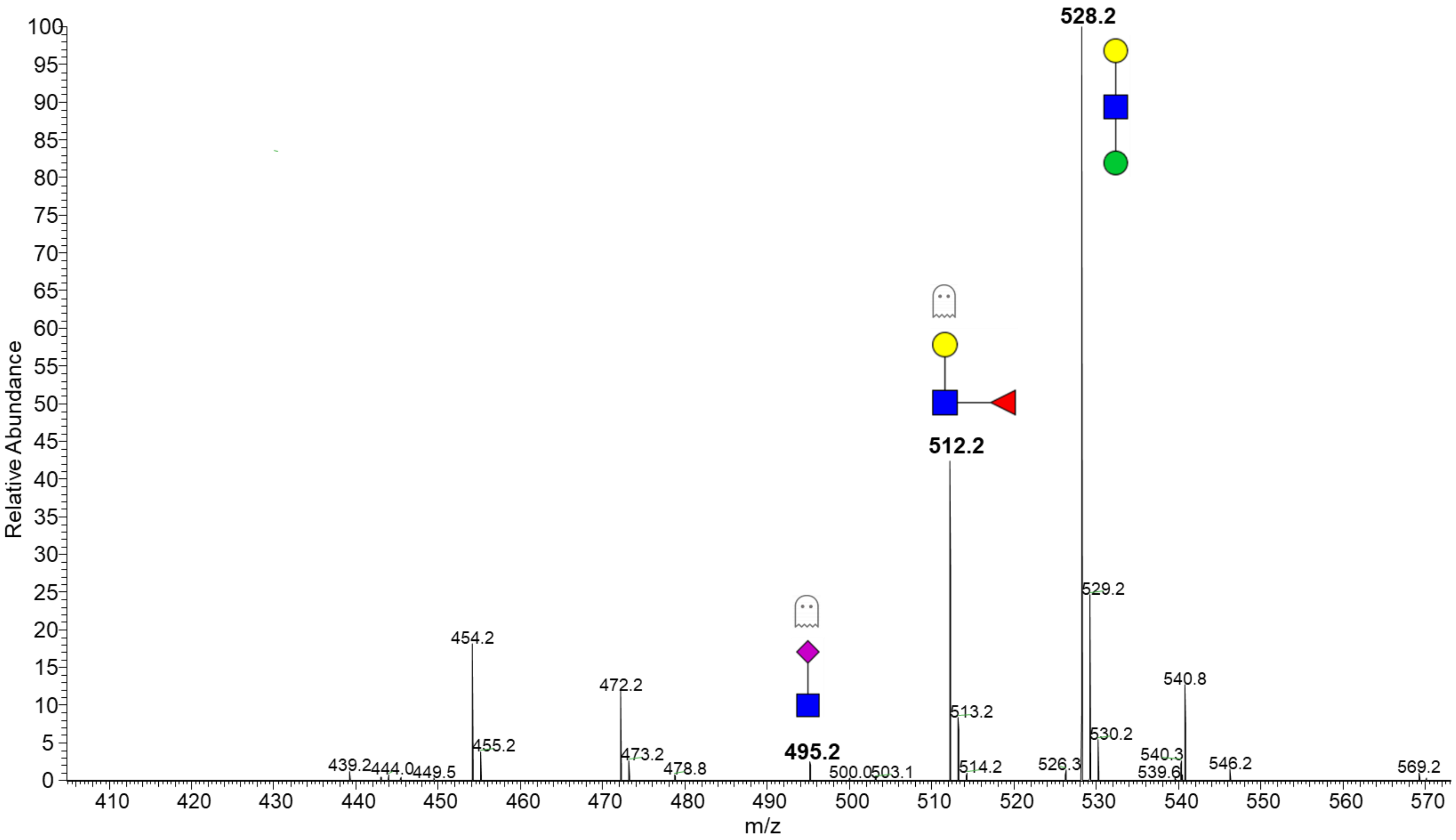

Figure S1. Example of ghost fragments in MS/MS spectra of G2FS IgG glycopeptide recorded under low collision energy NCE 15.

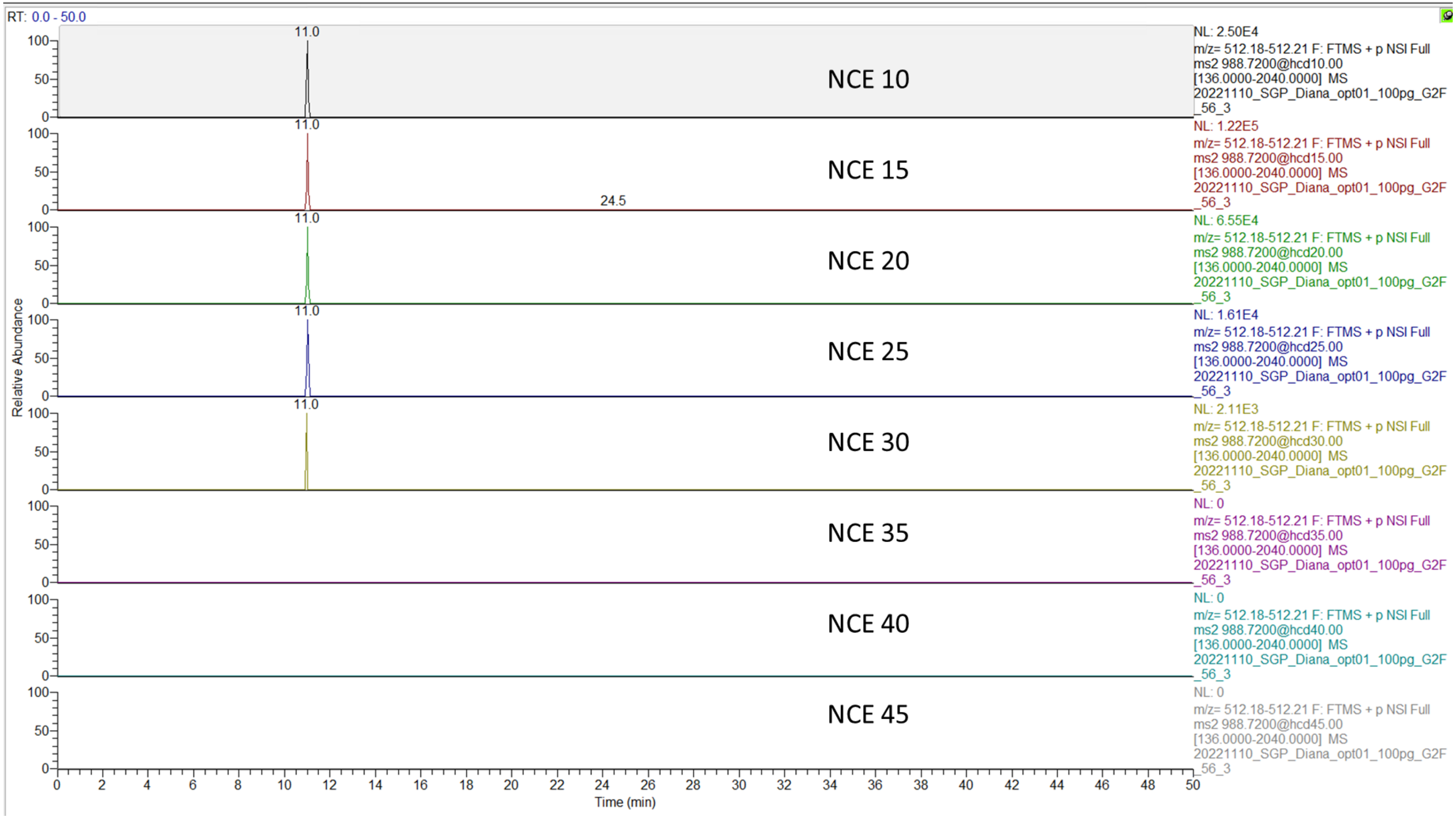

Figure S2. Chromatogram peaks under different NCE conditions (NCE10 to NCE45) of G2F standard for signal of LacNAc-fucose oxonium ion (m/z 512.2).

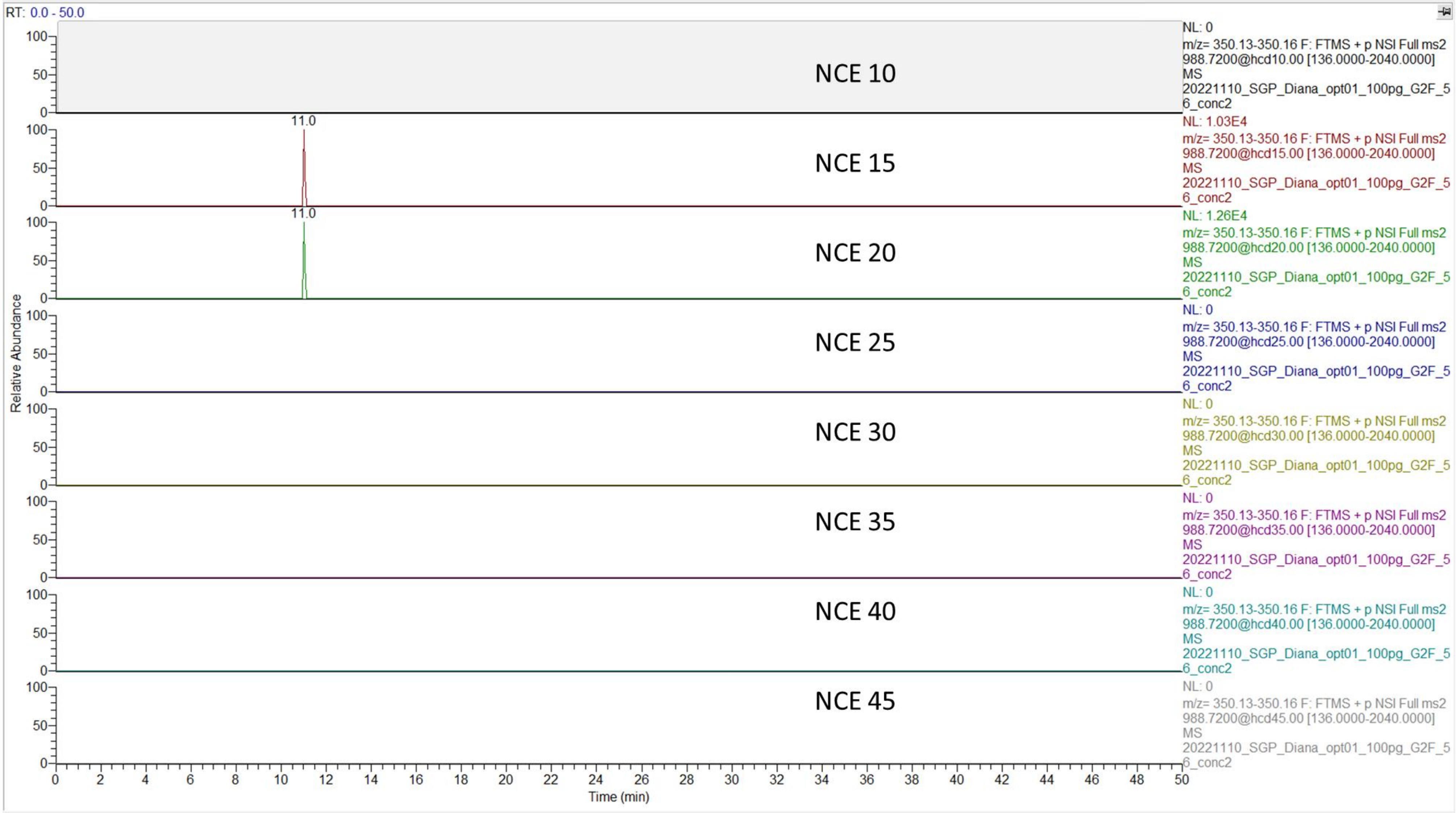

Figure S3. Chromatogram peaks under different NCE conditions (NCE10 to NCE45) of G2F standard for signal of GlcNAc-fucose oxonium ion (m/z 350.1).

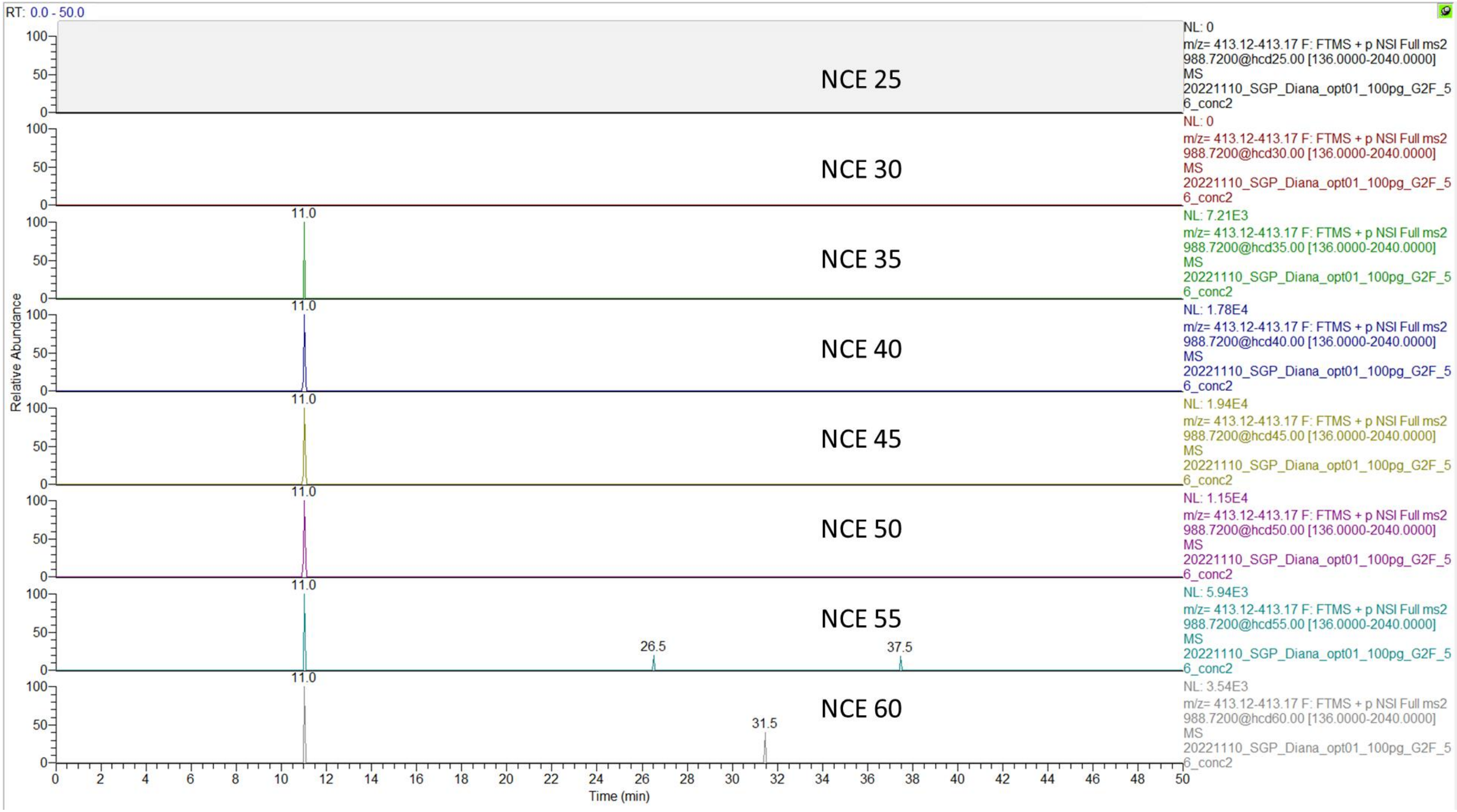

Figure S4. Chromatogram peaks under different NCE conditions (NCE25 to NCE60) of G2F standard for signal of LacdiNAc oxonium ion (m/z 413.2).

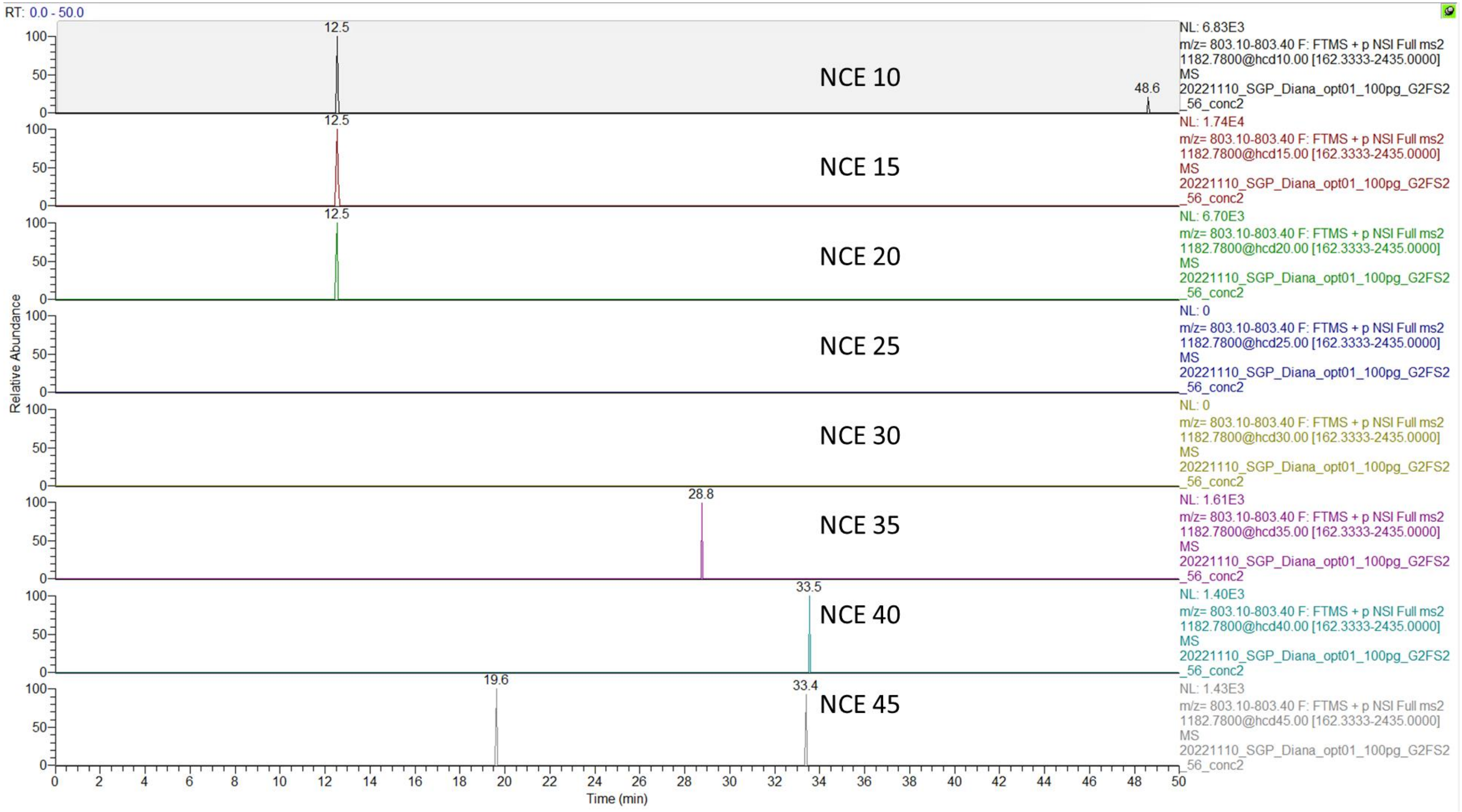

Figure S5. Chromatogram peaks under different NCE conditions (NCE10 to NCE45) of G2FS2 standard for signal of SialoLacNAc-fucose oxonium ion (m/z 803.3).

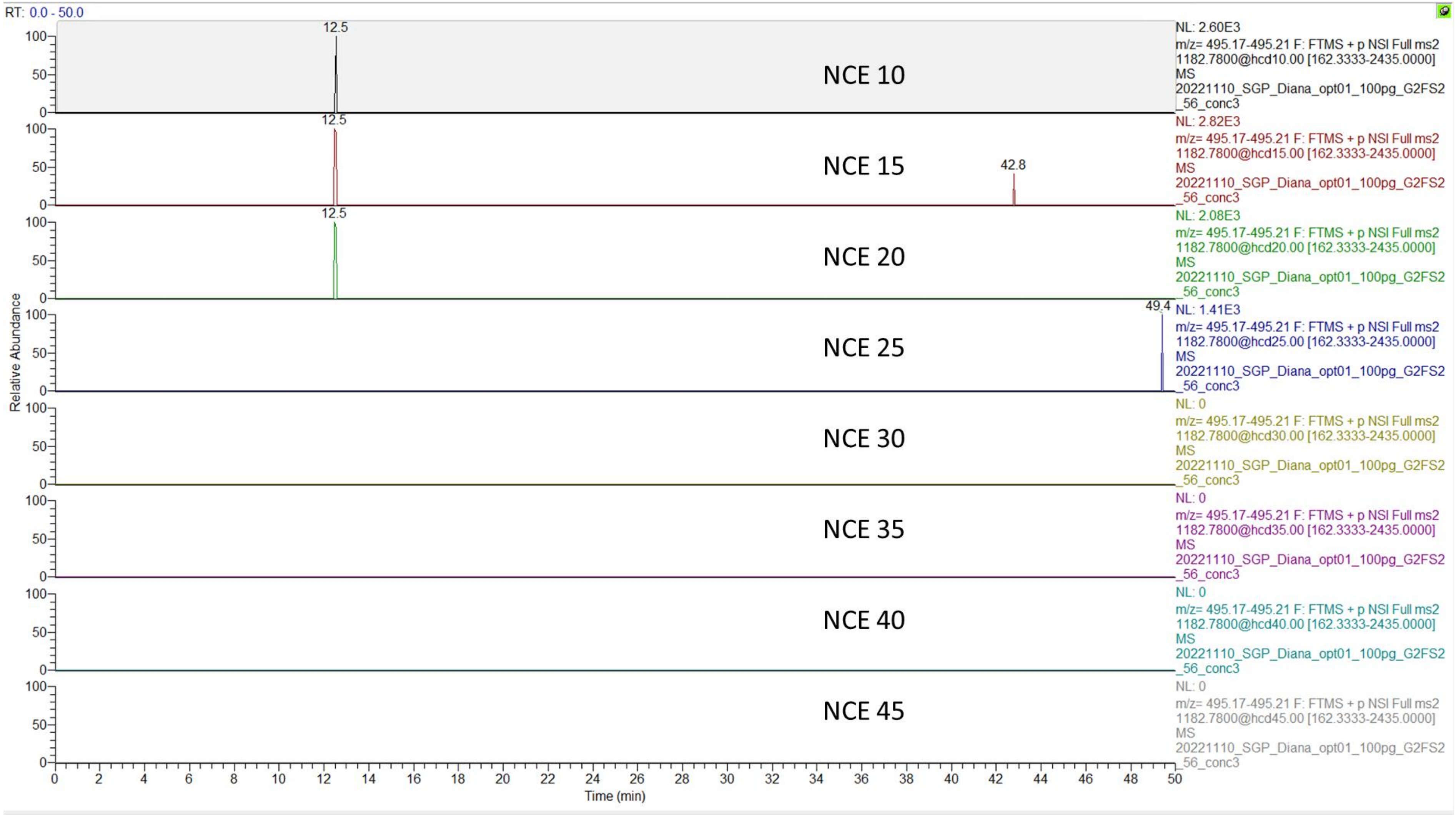

Figure S6. Chromatogram peaks under different NCE conditions (NCE10 to NCE45) of G2FS2 standard for signal of SialoLacdiNAc oxonium ion (m/z 495.2).

REFERENCES

(1) Sanda, M.; Yang, Q.; Zong, G.; Chen, H.; Zheng, Z.; Dhani, H.; Khan, K.; Kroemer, A.; Wang, L. X.; Goldman, R. LC-MS/MS-PRM Quantification of IgG Glycoforms Using Stable Isotope Labeled IgG1 Fc Glycopeptide Standard. *J Proteome Res* **2023**, *22* (4), 1138-1147. DOI: 10.1021/acs.jproteome.2c00475.

(2) Wang, S.; Liu, D.; Qu, J.; Zhu, H.; Chen, C.; Gibbons, C.; Greenway, H.; Wang, P.; Bollag, R. J.; Liu, K.; et al. Streamlined Subclass-Specific Absolute Quantification of Serum IgG Glycopeptides Using Synthetic Isotope-Labeled Standards. *Anal Chem* **2021**, *93* (10), 4449-4455. DOI: 10.1021/acs.analchem.0c04462.
